# Full-Length Transcriptome Sequencing Reveals the Molecular Mechanism of *Metasequoia glyptostroboides* Seed Responding to Aging

**DOI:** 10.3390/antiox12071353

**Published:** 2023-06-27

**Authors:** Yongjian Luo, Yixin Zhang, Jingyu Le, Qing Li, Jiaolin Mou, Shiming Deng, Jitao Li, Ru Wang, Zhijun Deng, Jun Liu

**Affiliations:** 1 Hubei Key Laboratory of Biologic Resources Protection and Utilization, Hubei Minzu University, Enshi 445000, China; 202030361@hbmzu.edu.cn (Y.L.); lejingyu_hbmzu@163.com (J.L.); moujiaolin@hbmzu.edu.cn (J.M.); dengshiming@hbmzu.edu.cn (S.D.); ljtyouth@foxmail.com (J.L.); ruwang_hbmzu@163.com (R.W.); 2 Guangdong Key Laboratory for Crop Germplasm Resources Preservation and Utilization, Agro-Biological Gene Research Center, Guangdong Academy of Agricultural Sciences, Guangzhou 510640, China; zhangyixin@agrogene.ac.cn (Y.Z.); caucbs07122@163.com (Q.L.); 3 Research Center for Germplasm Engineering of Characteristic Plant Resources in Enshi Prefecture, Hubei Minzu University, Enshi 445000, China; 4 The Plant Germplasm Resources Laboratory, School of Forestry and Horticulture, Hubei Minzu University, Enshi 445000, China

**Keywords:** ROS, oxidative phosphorylation, WGCNA, RBOH, PacBio-Sequence, germination percentage

## Abstract

*Metasequoia glyptostroboides,* Hu and W. C. Cheng, as the only surviving relict species of the Taxodiaceae *Metasequoia* genus, is a critically endangered and protected species in China. There is a risk of extinction due to the low vigor of *M. glyptostroboides* seeds, and the physiological mechanism of seed aging in *M. glyptostroboides* is not yet clear. In order to investigate the physiological and molecular mechanisms underlying the aging process of *M. glyptostroboides* seeds, we analyzed the antioxidant system and transcriptome at 0, 2, 4, 6, and 8 days after artificial accelerated aging treatment at 40 °C and 100% relative humidity. It was found that the germination percentage of fresh dried *M. glyptostroboides* seeds was 54 ± 5.29%, and significantly declined to 9.33 ± 1.88% after 6 days of aging, and then gradually decreased until the seed died on day 8. Superoxide dismutase (SOD) activity, ascorbic acid (AsA), glutathione (GSH) content and superoxide anion (O_2_^·−^) content and production rate significantly decreased, while malondialdehyde (MDA) and hydrogen peroxide (H_2_O_2_) content and glutathione peroxidase (GPX) and catalase (CAT) activity gradually increased during the aging process. A total of 42,189 unigenes were identified in the whole transcriptome, and 40,446 (95.86%) unigenes were annotated in at least one protein database. A total of 15,376 differentially expressed genes (DEGs) were obtained; KEGG enrichment analysis results revealed that seed aging may be mainly involved in the protein-processing pathways in endoplasmic reticulum, oxidative phosphorylation, and ascorbate and aldarate metabolism. Weighted gene co-expression network analysis (WGCNA) revealed that the dark magenta, orange, and medium purple modules were highly correlated with physiological indicators such as SOD, CAT, and GSH and further identified 40 hub genes such as *Rboh*, *ACO*, *HSF*, and *CML* as playing important roles in the antioxidant network of *M. glyptostroboides* seeds. These findings provide a broader perspective for studying the regulatory mechanism of seed aging and a large number of potential target genes for the breeding of other endangered gymnosperms.

## 1. Introduction

Seed vigor refers to the potential to determine the rapid and neat emergence of seeds and the normal growth of seedlings under field conditions; this was the main indicator used to measure seed quality and an important factor in determining yield [1]. Seed vigor reached its peak at late physiological maturity, after which it gradually and irreversibly declined during storage and transport, a change known as aging or deterioration [2]. During seed aging, a series of harmful changes occurred inside the seed, such as a decrease in the mechanical resistance of the seed coat, cell membrane damage, protein denaturation, DNA damage and mutation, and disruption of nucleic acid synthesis systems [3,4,5,6], uncontrolled reactive oxygen species’ (ROS) generation, and inefficient antioxidant machinery [7], resulting in a decline in germination percentage, seedling growth percentage and plant performance. The higher the degree of seed aging, the greater the vigor loss. Due to the long natural aging time of orthodox seeds, artificial accelerated aging or controlled deterioration treatment (CDT) is usually used in research to quickly obtain aging seeds through high temperature and humidity [8] or by breaking the balance of the seed cytoplasmic vitrification state (control temperature, water content, oxygen and other conditions) [9,10].

Recent advances in genomics and transcriptomics have provided new insights into the molecular mechanisms of seed aging. Multi-omics analysis has been used to identify genes related to seed vigor or aging resistance and their molecular mechanisms. Wang et al. identified that the transcription factor *bZIP23* bound to the genetic factor *PER1A* of peroxidase in rice through metabolome and transcriptome technologies, and was involved in the clearance of reactive oxygen species during seed aging [11]. Zhang et al. performed strand-specific RNA sequencing on rice embryonics of fresh-dried seeds (96% germination percentage) and aged seed samples (50% germination percentage; aged for 6 days at 43 °C and 85% relative humidity) and detected four upregulated lncRNAs that predominantly regulated target genes involved in base repair, either in cis or trans -regulated target genes [12]. Gu et al. conducted an integrated analysis of proteomics and genomics on 20 different rapeseed varieties with varying oil contents, identifying 165 differentially expressed proteins (DEPs), and 31 unique genes showed significant differences in the germination process between high- and low-oil-content seeds, with 13 of these genes potentially being related to seed germination vigor [13]. Rency et al. used QTL technology to discover eight genes related to the seed longevity mechanism, involved in functions such as reducing oxidative stress and repairing damaged DNA [14]. It was reported that acetaldehyde dehydrogenase 7 (*OsALDH7*) [15], Lipoxygenase 3 (*LOX3*) [16], OsGRETCHENHAGEN3-2 (*OsGH3-2*) [17], and protein repair L-isoaspartyl methyltransferase 1 (*OsPIMT1*) [18] were all associated with seed vigor and longevity in rice. Furthermore, research has shown that *OsHSP18.2* can protect and stabilize the structure and function of enzymes in cells by limiting the accumulation of ROS, thereby preventing irreversible damage [19]. Although there have been many advances in seed aging research in recent years, a large proportion of studies have focused on crop seeds, with less research being conducted on the mechanisms of seed aging in wild plants [20]. 

*Metasequoia glyptostroboides* was a plant species in the Cupressaceae family of the Metasequoia genus. It was a Quaternary glacial relic and was known as a “living fossil” in the plant kingdom [21]. The ancient history of *M. glyptostroboides* made it significant for studying ancient biology, climate, geology, and the systematic evolution of gymnosperms. However, wild populations of *M. glyptostroboides* had low genetic diversity and faced difficulties in natural regeneration. The age structure of the population was in an inverted pyramid shape, indicating a declining trend that showed no signs of improvement in its endangered status [22]. In recent decades, researchers have conducted much research on the physiological ecology of *M. glyptostroboides* seeds [23]. According to Li et al., the germination percentage of freshly harvested *M. glyptostroboides* seeds was only (32.9 ± 3.3)% [24], suggesting that the vigor of *M. glyptostroboides* seeds was relatively low, and under conditions of accelerated aging, the loss of seed vigor was exceptionally rapid compared to other plant species [25]. Liu et al. speculated that the low vigor and poor vigor retention ability of *M. glyptostroboides* seeds may be an important reason for the natural difficulty in updating its original population [25]. Therefore, it is imperative to further clarify the physiological mechanism underlying the loss of vigor in *M. glyptostroboides* seeds from a physiological perspective. This will facilitate a better understanding of the endangered mechanism of *M. glyptostroboides* and enable us to implement more effective measures to protect this species. 

Despite the rapid advances in sequencing technology, a genome map of *M. glyptostroboides* has not yet been published. The lack of high-quality genomic maps greatly limits research into plants with large genomes [8]. Full-length transcriptome sequencing belongs to the third-generation sequencing technology, which has advantages such as offering a comprehensive transcriptome analysis, improved gene annotation, precise quantification of transcripts, and spatial–temporal expression analysis [26]. For species without a reference genome, SMRT sequencing technology can significantly reduce the number of generated transcripts and provide a better understanding of the pathways related to whole transcriptomes, tissue development, and the secondary metabolism [8]. Therefore, this study, using full-length transcripts, explored the possible physiological mechanisms of the low vigor and rapid loss of vigor in *M. glyptostroboides* seeds. This will help us better understand the endangered mechanism of *M. glyptostroboides*, so as to better protect it, and may provide a reference for the protection of other endangered gymnosperms. 

## 2. Materials and Methods

### 2.1. Artificial Aging Treatment

During October 2020, fresh and mature seeds of *M. glyptostroboides* were collected in Lichuan, Hubei Province (E: 108°55′45.561″, N: 30°17′52.3″). After air-drying for 7 days, the seed water content was measured to be 10.06 ± 0.09% (*n* = 4) and the thousand-seed weight was 2.89 ± 0.06 g. Artificial aging treatment was conducted as in the work of Barreto and Garcia et al. [27] with minor modifications. The seeds were then placed in mesh bags and spread out in a constant-temperature and -humidity chamber with 40 °C and 100% relative humidity for 2 days (S2), 4 days (S4), 6 days (S6), and 8 days (S8). The aged seeds were frozen with liquid nitrogen and stored at −80 °C for subsequent experimental measurements. Fresh-dried and untreated seeds were used as the control (S0).

### 2.2. Germination Parameters Tests

Germination tests were conducted according to International Seed Testing Association (ISTA, 2018) criteria, and 3 replicates were used, with around 50 seeds per biological replicate [28]. The seeds were evenly seeded in a petri dish (diameter: 9 cm) covered with 2 layers of filter paper, and then placed in a 25 °C constant-temperature light incubator (12 h light/12 h dark, PPFD = 121 μmol m^−2^s^−1^) with distilled water added [29]. A seed was considered to be germinated if it developed into a normal seedling. Germination percentage was the normal seedling percentage at the final day (day 16). Leaf length (LL), root length (RL) and seedling fresh weight (FW) were also measured on 14th day. Seeds with primary roots of at least 2 mm long were recorded every day until the 10th day to calculate mean germination time (MGT) using Equation (1):

MGT = ∑NT/∑N(1)

Vigor index (VI) was calculated according to Equation (2):

VI = ∑(G_T_/T) × FW (2)

where T is the number of days counted from the beginning of germination, N is the number of seeds germinated on day T, and G_T_ is the number of germinated seeds per day corresponding to T. The data were calculated and presented as the mean ± SD of three biological replicates, and subjected to ANOVA to determine significant differences.

### 2.3. Physiological Analysis

According to the detection protocol provided by Suzhou Keming Biotechnology Co., LTD. (Suzhou, China) (www.cominbio.com, accessed on 15 February 2023), The antioxidant enzyme activities of seeds’ superoxide dismutase (SOD, EC 1.15.1.1), glutathione peroxidase (GPX, EC 1.11.1.9), ascorbic acid peroxidase (APX, EC 1.11.1.11), catalase (CAT, EC 1.11.1.11)] were detected by kits. EC 1.11.1.11)], as well as the contents of O_2_^·−^, malondialdehyde (MDA), H_2_O_2_, glutathione (GSH), L-ascorbic acid (AsA) and O_2_^·−^ production rate. The data were calculated and presented as mean ± SD of three biological replicates, then subjected to ANOVA to determine significant differences. 

### 2.4. RNA Quantification and Qualification 

The RNA for Pacbio full-length transcriptome sequencing was extracted from a mixed sample of *M. glyptostroboides* root, stem, leaves, and seeds, while the RNA sample for Illumina sequencing was obtained from *M. glyptostroboides* seeds at different stages of aging. The Plant Total RNA Purification Kit (TSINGKE, Beijing, China) was used to extract RNA from *M. glyptostroboides* tissues, and then the RNA integrity was measured using Agilent 2100 Bioanalyzer (Supplementary Figure S1) (Agilent Technologies, Santa Clara, CA, USA) and the RNA concentration was estimated using Qubit Fluorometer (Thermo Fisher Scientific, Waltham, MA, USA). 

### 2.5. cDNA Construction and PacBio Iso-Seq

Following the PacBio Isoform Sequencing protocol, cDNA synthesis and library construction were performed using the Clontech SMARTer PCR cDNA Synthesis kit (Clontech, Mountain View, CA, USA). The full-length cDNA Iso-Seq templates of 2 SMRT were sequenced after purification, size selection, re-amplification and SMRTbell template preparation. The cDNA library was size-selected using BluePippin (Sage Science, Beverly, MA, USA) to include only cDNAs larger than 4 kb. Both cDNAs larger than 4 kb and non-selected cDNAs were combined in equal amounts to form the Iso-Seq library for SMRT sequencing.

### 2.6. Iso-Seq Data Processing with Standard Bioinformatics Pipeline 

The raw sequencing data underwent processing using SMRTlink4.0 software, following the standard Iso-Seq protocol. Initially, reads of insert (ROIs) were generated by liminating adapters and artifacts from subreads. These ROIs were then classified into two groups, full-length nonchimeric (FLNC) reads and non-full-length (nFL) reads, by detecting primer and polyA tail with ‘pbclassify.py’. Clustering of the FLNC reads was carried out using the iterative clustering for error correction (ICE) algorithm. Subsequently, the consensus sequences obtained were polished and categorized using the nFL reads with the Quiver algorithm, followed by error correction using the ‘proofread (2.13.12)’ tool and an Illumina RNA-seq dataset. Finally, redundancies were eliminated using CD-hit to obtain the final set of non-redundant full-length transcripts.

The obtained unigenes were subjected to annotation analysis by mapping them to seven databases. For nucleotide database (Nt) analysis, we utilized the BLAST software with an e-value threshold of ‘1 × 10^−5^’. In the protein family database (Pfam) analysis, the Hmmscan software was employed. The non-redundant protein database (Nr), Kyoto Encyclopedia of Genes and Genomes (KEGG), cluster of orthologous group (KOG), and Swiss-Prot, and Gene Ontology (GO) databases were annotated using BLASTX with a cut-off e-value of ‘1 × 10^−5^’.

### 2.7. Illumina Library Construction and Sequencing 

Seeds that underwent aging treatment were collected and subjected to total RNA extraction. The extracted RNAs were then sequenced using the Illumina HiSeq 2500 platform (Illumina Inc., Foster City, CA, USA) with three biological replicates. The resulting sequences were aligned to the reference full-length transcript of *M. glyptostroboides* constructed in this study. The number of fragments mapped to each transcript was tallied to determine the relative expression level of each transcript. The expression levels of genes were normalized using the fragments per kilobase of transcript per million mapped reads (FPKM) method. Genes exhibiting a fold change ratio of ≥2, with a corrected *p*-value < 0.05, were identified as DEGs.

### 2.8. Weighted Correlation Network Analysis

Weighted gene co-expression network analysis (WGCNA) was carried out with the WGCNA package (v1.72-1) in R [30]. The co-expression analysis was conducted on 15 samples. The DEGs were assigned to 15 modules using WGCNA. Correlations between each module and seed-aging stress were calculated. Genes at different expression levels were assigned to various modules via a dynamic tree cut. There were at least 30 genes per co-expression module. Certain modules were similar. Correlations among various modules were calculated using 0.25 as the similarity threshold. Module-trait associations were estimated from the correlations between the module eigengene and various seed-aging stages or enzyme activity levels. Each node represented one gene connected to several others. The node size was proportional to the number of genes to which a specific gene was linked. The total connectivity and intramodular connectivity (function soft connectivity), modular membership (kME), and kME *p* values were calculated for the DEGs.

### 2.9. Quantitative (q)RT-PCR Validation

The RNA samples isolated above were used as templates and were reverse-tran-scribed with a PrimeScript™ RT reagent Kit (Takara, Beijing, China). The primers used in this study were designed via Primer 5 with RefSeq and are listed in Supplementary Table S1. The expression of the beta-actin gene was used as an internal control. qRT-PCR was performed with Luna^®^ Universal qPCR Master Mix (NEB, Beijing, China) on a QuantStudio™ 5 device (Thermo Fisher, USA) according to the manufacturercs’ protocol. Relative gene expression levels were evaluated according to the 2^−ΔΔCT^ method [31]. 

## 3. Results

### 3.1. Germination Percentage and Physiological Changes during Artificial Seed Aging

**Figure 1:**
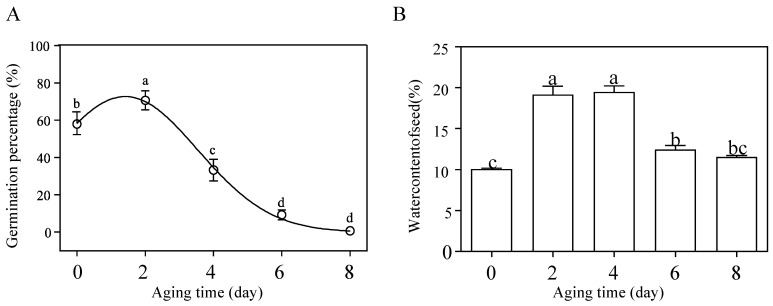
Dynamic map of germination percentage and water content of *M. glyptostroboides* seeds during artificial aging treatment. (**A**) Changes in seed germination percentage with artificial aging treatment time. (**B**) Changes in seed water content with artificial aging treatment time. Note: Different letters on top of each bar indicate statistically significant differences (*p* < 0.05).

The germination percentage of *M. glyptostroboides* seeds was investigated after artificial aging treatment. The results showed that the germination percentage after 2 days of aging treatment was 70.67 ± 3.68%, which was significantly different from the control group, at 58.00 ± 5.71%. Then, the germination percentage exhibited a continuous downward trend, and was almost zero after 8 days of aging (Figure 1A). The water content of *M. glyptostroboides* seeds showed an initial increase followed by a decreasing trend (Figure 1B). The water content of fresh seeds without aging treatment was 10.06 ± 0.13%. The seeds aging for 2 and 4 days significantly increased the water content to 19.15 ± 1.04% and 19.49 ± 0.76%, respectively. However, with further aging, the seed water content began to significantly decrease at 6 days of aging and decreased to 11.54 ± 0.20% at 8 days of aging. The germination potential and germination index of *M. glyptostroboides* seeds decreased with the increase in aging time, and the vigor index, root length, seedling height, fresh weight, and dry weight showed the same trend as the germination percentage (Table 1). Root length, seedling height, and fresh weight showed no significant difference from the control group after 2, 4, and 6 days of aging. Dry weight also showed no significant difference from the control group after 2 and 4 days of aging, and only showed a significant decrease after 6 days of aging.

**Figure 2:**
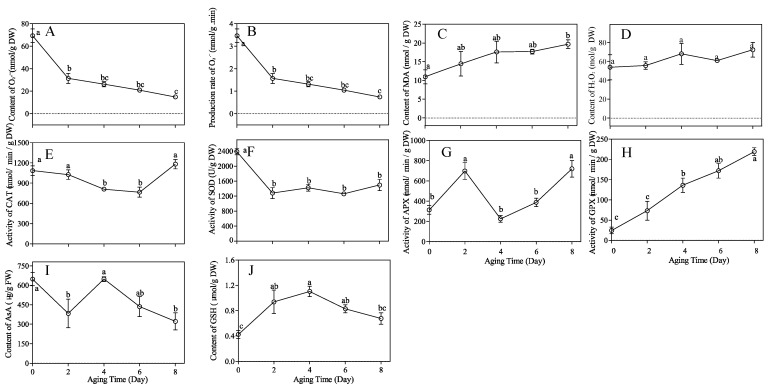
Determination of different physiological indices of the *M. glyptostroboides* seeds under aging treatment, (**A**) O_2_^·−^ content, (**B**) O_2_^·−^ production rate, (**C**) MDA content, (**D**) H_2_O_2_ content, (**E**) CAT activity, (**F**) SOD activity, (**G**) APX activity, (**H**) GPX activity, (**I**) AsA content, (**J**) GSH content. The data are shown as the means ± SDs of three independent experiments. Note: Different letters on top of each bar indicate statistically significant differences (*p* < 0.05).

The artificial aging treatment had a significant effect on the content and production rate of superoxide anion radicals (O_2_^·−^) in *M. glyptostroboides* seeds. With the increase in aging time, the content and production rate of O_2_^·−^ showed a decreasing trend, with a faster decline rate at the beginning and a slower rate later (Figure 2A,B). The content of malondialdehyde (MDA) in seeds was gradually upregulated with the increase in aging time, and the difference was significant at the S8 stage compared with the S0 stage (Figure 2C). The H_2_O_2_ content did not change significantly with the treatment time (Figure 2D). The activities of the four main antioxidant enzymes, superoxide dismutase (SOD), catalase (CAT), ascorbate peroxidase (APX), and glutathione peroxidase (GPX), showed significant changes. SOD activity decreased rapidly to a lower level after 2 d aging and then remained relatively stable at a low level (Figure 2F); CAT activity showed a trend of first decreasing and then increasing with aging (Figure 2E); APX activity showed a trend of “increase-decrease-increase” with aging time (Figure 2G); GPX activity continued to increase with aging time (Figure 2H). As for non-enzymatic antioxidants, the ascorbic acid (AsA) content, showed a “decrease-increase-decrease” trend. The AsA content in the control group reached its maximum value and was not significantly different from that of the 4d aging group, but was significantly higher than that of other aging timepoints (Figure 2I). In addition, the glutathione (GSH) content showed an “increase-decrease” trend (Figure 2J). On the 8th day, the GSH content in the seeds was not significantly different from that of the control group, while the GSH content in the seeds under other treatments was significantly higher than that of the control group.

### 3.2. Functional Annotation of M. glyptostroboides Transcriptome

**Figure 3:**
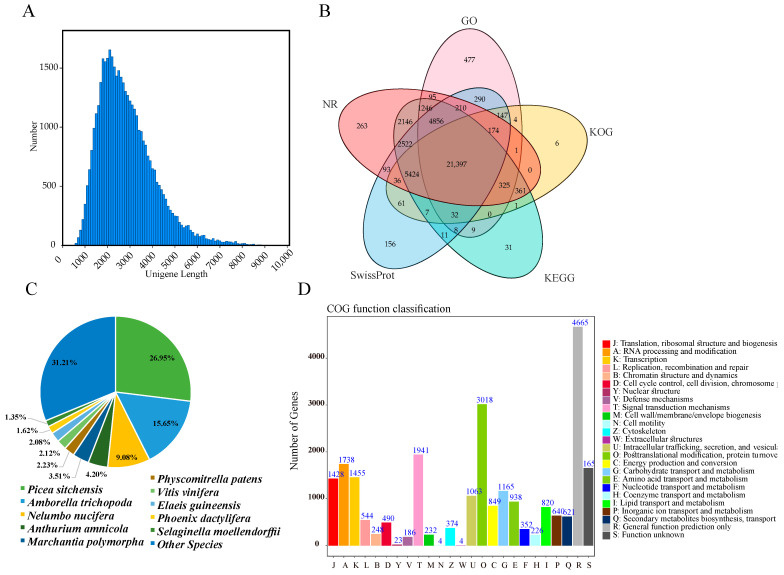
Overview of SMRT sequencing results and annotation of unigenes. (**A**) Distribution of full-length reads. (**B**) Overlap between the number of all unigenes according to five databases. (**C**) Distribution of unigene annotations based on the NR database for the species distribution statistics. (**D**) KOG functional classification of all unigenes.

The NGS transcriptome sequencing generated a total of 636,418,458 raw reads and 613,316,468 clean reads (96.37%, 91.25 Gb), with an average Q30 value of 93.70% and an average GC content of 45.54% (Table S2). The SMRT transcriptome sequencing generated 11,487,282 subreads (32.18 Gb in size), with an average length of 2802 bp and an N50 value of 3108 bp. Among these subreads, 289,659 full-length nonchimeric (FLNC) reads were obtained, with an average length of 3084 bp, which were corrected using the NGS data (Figure 3A). A total of 330,124 circular consensus sequences (CCSs) were obtained after filtration of the subreads. The FLNC sequences of the same transcript were clustered, and redundancies were removed using the hierarchical n*log(n) algorithm, resulting in 77,558 consensus sequences. CD-HIT was used to remove redundancies in the consensus sequences, ultimately obtaining 42,189 unigenes with an average length of 2814 bp (Figure 3A).

To determine the possible functions of unigenes in *M. glyptostroboides*, a total of 42,189 unigenes in the SMRT transcriptome were functionally annotated using five databases (the NR, GO, KEGG, KOG, and SwissProt databases). All the unigenes had an approximately 95.86% annotation rate in at least one database (NR (39,148, 92.79%), GO (29,270, 69.38%), KEGG (38,375, 90.96%), KOG (27,975, 66.31%), and SwissProt (35,423, 83.96%)) (Figure 3B, Supplementary Table S3). To determine the conservation level of the unigene sequences of *M. glyptostroboides* in other plant species, the unigene sequences in *M. glyptostroboides* were queried in the NCBI NR database (Figure 3C). The unigene sequences in *M. glyptostroboides* displayed the highest similarity to sequences from *Picea sitchensis* (10,532, 26.95%), followed by those from Amborella trichopoda (6113, 15.65%), Nelumbo nucifera (3546, 9.08%), Anthurium Amnicola (1641, 4.20%) and Marchantia polymorpha (1370, 3.51%). All the *M. glyptostroboides* unigenes were subsequently queried against the KOG database (Figure 3D). A total of 27,975 sequences were annotated, with 26 functional categories. ‘Posttranslational modification, protein turnover, and chaperones’ (3018, 12.22%); ‘general function prediction only’ (2593, 16.64%); and ‘signal transduction mechanisms’ (1941, 7.86%) were the top three categories. Furthermore, ‘Cell motility and Extracellular structures’ (4, 0.0094%) was the smallest category with the fewest unigenes. A total of 29,270 unigenes were annotated in the GO database (Supplementary Table S4), which were successfully clustered into 53 functional groups, of which 25 categories belonged to biological processes (BPs), 18 belonged to cellular components (CCs), and 10 belonged to molecular functions (MFs). We also conducted an analysis based on KEGG pathways to obtain key information, including intracellular metabolic pathways and biological functions of genes in *M. glyptostroboides* (Supplementary Table S4). A total of 38,375 unigene sequences were clustered into 19 KEGG pathway categories. Moreover, the most significant category among these pathways was ‘Signal transduction’ (2250; 5.86%), followed by ‘translation’ (1882; 4.90%) and then ‘carbohydrate metabolism’ (1767; 4.60%).

### 3.3. Global Analysis of the Time-Course Transcriptome Data from Different Samples

**Figure 4:**
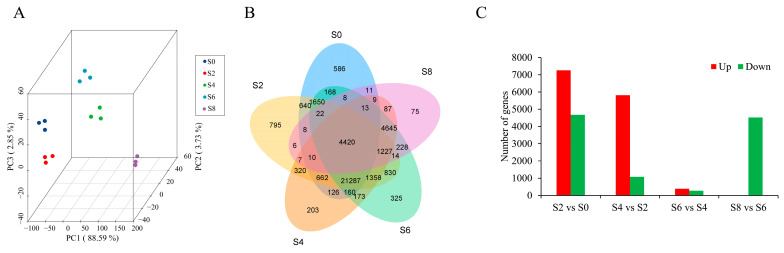
Transcriptome gene expression statistics and differential expression analysis. (**A**) PCA plot of transcriptome gene expression in different samples. (**B**) Venn diagram of DEGs in each sample. (**C**) Differential expression analysis between different samples.

We performed expression quantification analysis using RSEM software with unigenes as reference sequences, based on five stages of RNA-seq with three replicates, generating a total of 36–47 million clean reads, and aligned to 46.75–84.23% unigenes (Supplementary Table S5). Then, we calculated Fragments Per Kilobase Million (FPKM) for each gene based on its length and performed a PCA analysis on 15 samples based on FPKM, revealing good repeatability among replicates and significant differences among different groups (Figure 4A). A total of 40,096 unigenes with FPKM > 0.3 were detected in all 15 samples (Figure 4B). By comparing the expression levels of five aging stages of seeds, we found that the gene expression in *M. glyptostroboides* seeds on the 8th day of aging was significantly lower than that in the CK group, with a large number of genes rapidly decreasing to near zero, indicating seed death and DNA degradation (Supplementary Figure S2). Differential expression analysis revealed that the largest number of DEGs in *M. glyptostroboides* seeds was observed in the S2 vs. S0 group, the S6 vs. S4 group had the fewest DEGs, and all the DEGs were downregulated in the S8 vs. S6 group (Figure 4C).

**Figure 5:**
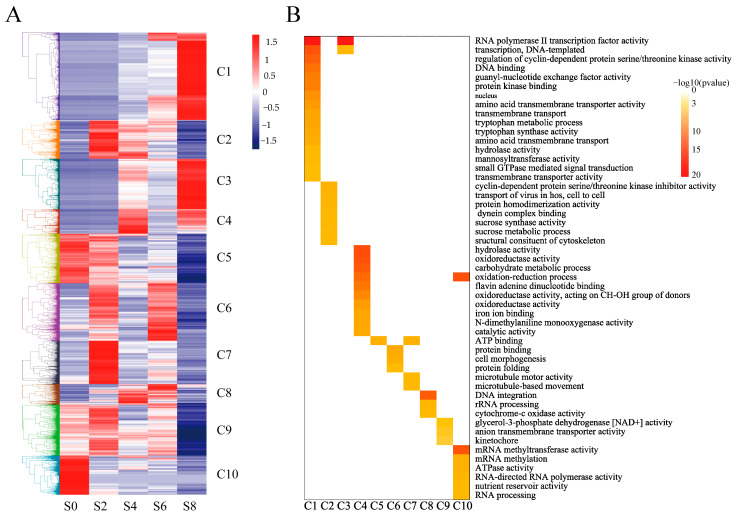
Differential expression gene temporal analysis. (**A**) Clustering heatmap of DEGs at different treatment times. (**B**) GO functional enrichment analysis of different gene clusters.

In order to investigate the expression pattern and functions of these DEGs during seed aging, the DEGs were analyzed by k-means clustering. The k-means clustering analysis revealed 10 distinct clusters, named C1–C10. The results of different gene clusters showed different trends over time (Figure 5A). Functional enrichment analysis of different cluster modules showed that gene clusters C1, C3, and C4 were gradually upregulated with seed aging, and their gene functions were involved in the oxidation-reduction process, tryptophan metabolic process, carbohydrate metabolic process, and other functions. These genes may play important roles during seed aging. Gene clusters C5 and C10 were gradually downregulated with seed aging, and their gene expression functions were involved in mRNA methylation, RNA processing, and other functions. Gene clusters C2, C6, and C9 showed upregulation followed by downregulation, and their gene functions were involved in protein folding, sucrose metabolic process, and other biological functions. The functions of gene clusters C8 and C7 were involved in rRNA processing, DNA integration, microtubule motor activity, etc.

### 3.4. GO and KEGG Functional Enrichment Analysis 

**Figure 6:**
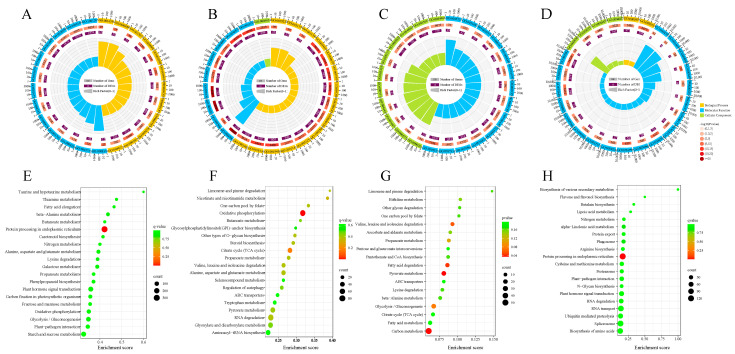
GO and KEGG functional annotation of DEGs at different stages. (**A**–**D**) The results of the GO term enrichment analysis of the group S2 vs. S0, S4 vs. S2, S6 vs. S4 and S8 vs. S6. DEGs were presented in the ‘TreeMap’ view of REVIGO. Each rectangle represents a single cluster representative, and the representatives are grouped into ‘superclusters’ of loosely related terms, visualized with different colors. The size of the rectangles was adjusted to reflect the *p*-value. (**E**–**H**) The results of KEGG enrichment analysis of the group S2 vs. S0, S4 vs. S2, S6 vs. S4 and S8 vs. S6. DEGs are presented.

GO and KEGG enrichment analysis of DEGs showed that in the S2 stage (S2 vs. S0), a total of 3416 GO terms were enriched; among them, 266 GO terms were significantly enriched such as oxidoreductase activity (GO:0016491), carbohydrate metabolic process (GO:0005975), and response to oxidative stress (GO:0006979). (Figure 6A, Supplementary Table S6). In the S4 stage (S4 vs. S2), 142 GO terms such as the regulation of protein modification process (GO:0031399), phospholipid catabolic process (GO:0009395), oxidoreductase activity (GO:0016491), and DNA-directed RNA polymerase complex (GO:0006366) were significantly enriched (Figure 6B, Supplementary Table S7). In the S6 stage (S6 vs. S4), 22 GO terms such as DNA-templated transcription (GO:0006353), regulation of cytokinesis (GO:0032465), and beta-galactosidase complex (GO:0004565) were significantly enriched (Figure 6C, Supplementary Table S8). In the S8 stage (S8 vs. S6), 95 GO terms such as protein–tetrapyrrole linkage (GO:0017006), regulation of developmental process (GO:0050793), unfolded protein binding (GO:0051082), protein kinase CK2 complex (GO:0005956), ubiquitin–protein transferase activity (GO:0004842), and oxidoreductase activity (GO:0016307) were significantly enriched (Figure 6D, Supplementary Table S9). The KEGG enrichment analysis of DEGs showed that in the S2 stage (S2 vs. S0) and S8 stage (S8 vs. S6), the DEGs were mainly enriched in pathways such as protein processing in endoplasmic reticulum, and plant hormone signal transduction. (Figure 6E,H). In the S4 stage (S4 vs. S2), the DEGs were significantly enriched in pathways such as oxidative phosphorylation, RNA degradation, and limonene and pinene degradation. (Figure 6F). In the S6 stage (S6 vs. S4), the DEGs were significantly enriched in pathways such as ascorbate and aldarate metabolism, and fatty acid degradation. (Figure 6G). 

### 3.5. Expression Analysis of Genes Associated with the Protein Processing in Endoplasmic Reticulum Pathway

**Figure 7:**
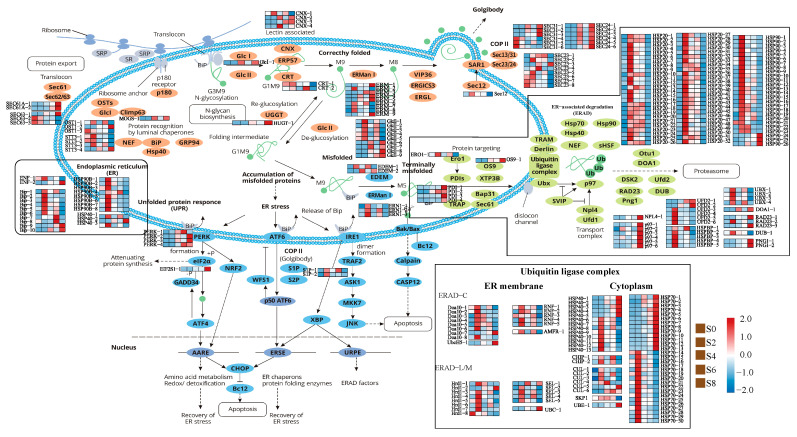
DEGs related to protein processing in endoplasmic reticulum pathways during artificial aging treatment of *M. glyptostroboides* seeds.

A total of 348 DEGs related to endoplasmic reticulum stress (ERS) were found by functional annotation (Figure 7, Supplementary Table S10), among which *BiP* (binding protein) is an important molecular chaperone protein and a member of the heat shock protein (HSP) 70 family. The expression of *BiP* was significantly upregulated under artificial aging treatment, suggesting that ERS occurred, and the number of unfolded and misfolded proteins increased during the aging process. Among the DEGs related to ERS, four calnexin (*CNX*) genes were significantly upregulated, and the glucose-regulated protein 94 gene (*GRP94*), which encoded a molecular chaperone, was also upregulated during S2 and S8 phases. It was also found that DEGs involved in protein processing in the endoplasmic reticulum pathway were mainly concentrated in the endoplasmic reticulum-associated degradation (ERAD) pathway. A total of 201 DEGs were detected to participate in three processes of ERAD pathway, including substrate protein recognition, transport, and ubiquitination, followed by proteasomal degradation. Among them, nine mannosyl-oligosaccharide alpha-1,2-mannosidase (*ERManl*) genes and six transitional endoplasmic reticulum ATPase (*p97*) genes were upregulated in the S8 phase. In the transport process, the expression of protein disulfide-isomerase (*PDI*) and ER degradation enhancer (*EDEM*), which promoted substrate release, were upregulated, and the genes encoding related enzymes that transfer substrate proteins to the ubiquitin ligase complex, such as protein OS-9 (*OS-9*) and ERO1-like protein alpha (*ERO1*), were also upregulated. Hsps belonged to the molecular chaperone protein family that bound to unfolded or misfolded proteins to promote the correct folding of nascent proteins. There were 134 *HSP* family members, among which 105 *HSP* family members were highly expressed in the S2 phase and then gradually decreased. It was worth noting that there were 13 members of *HSP70* and 5 members of *HSP40* family proteins, with the highest expression in the S8 phase, which participated in the ubiquitin ligase complex. There were 21 DEGs encoding components of the ubiquitin ligase complex, and the expression levels were generally upregulated after 2 days of aging, with some genes such as *p97*, peptide-N4-(N-acetyl-beta-glucosaminyl) asparagine amidase (*PNG*), and Ubiquitin C (*UBC*) showing the highest expression levels in the S8 phase. 

### 3.6. Expression Analysis of Genes Associated with the Oxidative Phosphorylation Pathway

**Figure 8:**
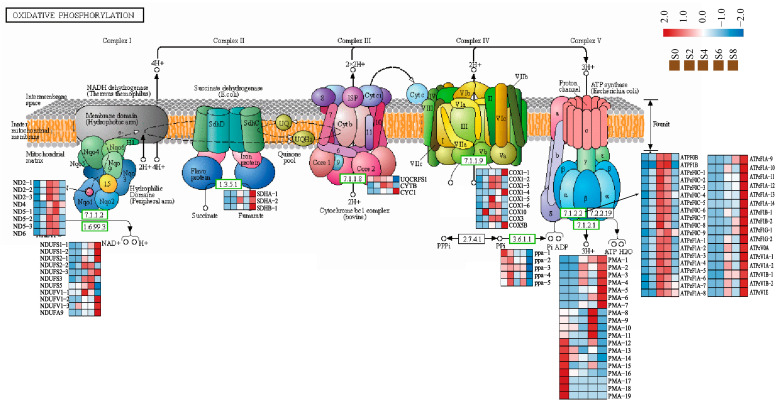
DEGs related to the oxidative phosphorylation pathways during artificial aging of *M. glyptostroboides* seeds.

Mitochondria were the main site of energy metabolism in eukaryotes, playing an important role in physiological and pathological activities such as free radical production, cell apoptosis, and aging, and were an important target organelle of oxidative stress. In this study, a total of 93 DEGs were identified in the oxidative phosphorylation pathway during seed aging in *M. glyptostroboides* (Figure 8, Supplementary Table S11). Among them, cyclooxygenase (*COX*), a marker of seed stress, was expressed at low levels in non-aged seeds, but was significantly upregulated from S2 to S4, and downregulated as aging progressed. *ATPase* was the most abundant with 35 genes of 9 types, and the overall expression trend was significantly upregulated after 2 days of aging. Four kinds of NADH-Ubiquinone oxidoreductase chain and six kinds of NADH dehydrogenase (ubiquinone) Fe-S genes are identified in complex I on the mitochondrial electron transport chain, and their expression trend was generally upregulated after seed aging.

### 3.7. Effects of Aging Stress on the Antioxidant Responses

**Figure 9:**
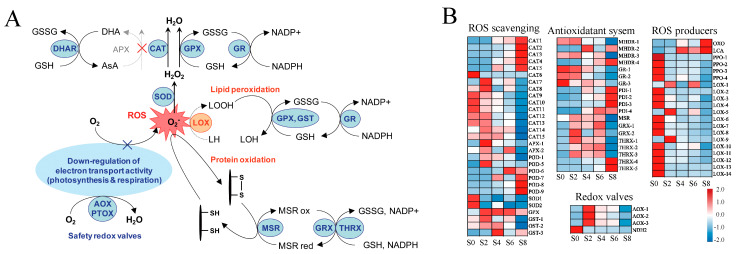
Production and elimination of reactive oxygen species during seed aging. (**A**) Production and elimination of ROS. (**B**) The identified candidate genes involved in ROS scavenging system in response to seed aging.

During seed storage, the accumulation of ROS led to lipid damage, DNA and protein degradation, resulting in reduced germination percentage and loss of seed vigor [32]. The ROS scavenging system in higher plants is mainly composed of the ascorbate–glutathione (AsA-GSH) cycle pathway, the glutathione peroxidase GPX pathway, the catalase CAT pathway, and the peroxiredoxin/thioredoxin (PrxR/Trx) pathway [33] (Figure 9A). Therefore, DEGs related to these pathways were analyzed (Figure 9B, Supplementary Table S12). A total of 32 DEGs were detected in the ROS scavenging system, including 15 CAT, 2 APX, 9 POD, 2 SOD, 1 GPX, and 3 GST. Among them, the expression of 10 *CAT* was downregulated with aging and 5 were upregulated, showing an overall downregulation trend. The expression of 9 *POD* was upregulated with aging time. In the antioxidant system, *GR* was gradually downregulated with the duration of aging, while other genes such as *PDI* and *THRX* were gradually upregulated. Lipoxygenase (*LOX*), a key enzyme in lipid peroxidation, was significantly downregulated after aging. Combined with the upregulated expression of most *TRX*, *GST*, and *GSH* genes in *M. glyptostroboides* seeds after aging, and the downregulated expression of most *LOX* genes after aging treatment, it can be seen that the antioxidant system of *M. glyptostroboides* seeds alleviated the process of lipid peroxidation to some extent, so the MDA content showed an upward trend but did not change significantly. In combination with the fact that most *TRX*, *GST*, and *GSH* genes were upregulated in expression after aging treatment, while most *LOX* genes were downregulated in expression after aging treatment, it was evident that, to some extent, the antioxidant system of *M. glyptostroboides* seeds alleviated the lipid peroxidation process; thus, the MDA content did not change significantly, although it showed an increasing trend. It is worth noting that the changes in the expression levels of *APX*, *CAT*, and *GSH* genes are consistent with the results of the physiological indicators mentioned earlier, except for the *GPX* gene.

### 3.8. Co-Expression Network Analysis of DEGs by WGCNA

**Figure 10:**
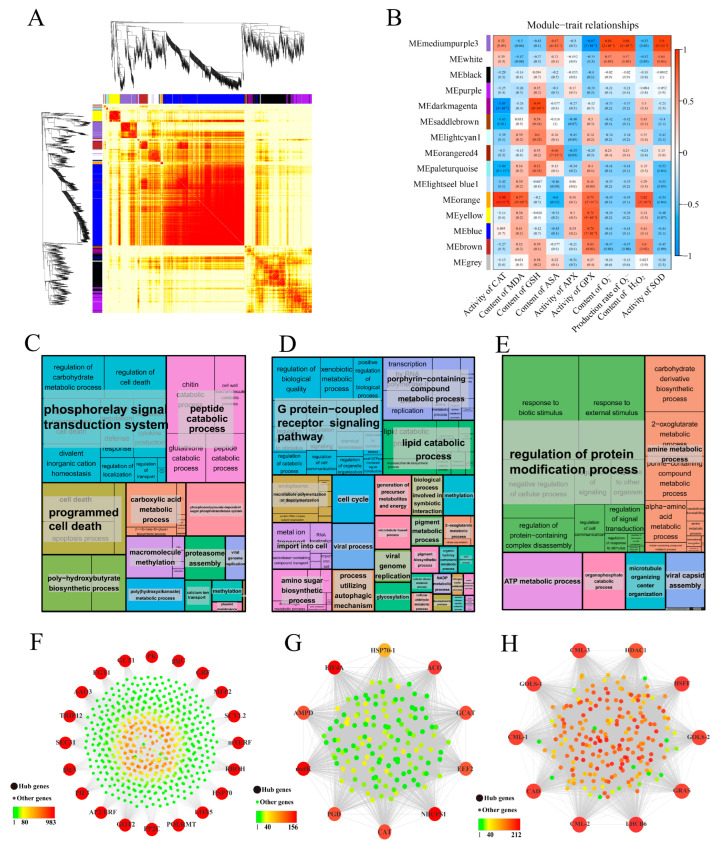
WGCNA of the identified genes during *M. glyptostroboides* seeds aging. (**A**) Gene dendrogram obtained by clustering the dissimilarity based on consensus topological overlap, with each tree branch constituting a module and each leaf representing one gene. Each colored row indicates a color-coded module that contains a group of highly interconnected genes. (**B**) Module eigengene physiological indexes and sample correlations. (**C**–**E**). The results of the GO term enrichment analysis of the medium purple 3, orange and dark magenta module genes visualized by the ‘TreeMap’ view of REVIGO. Each”rect’ngle is representative of a single cluster. The representatives are joined into ‘superclusters’ of loosely related terms, visualized with different colors. The size of the rectangles is adjusted to reflect the *p*-value. (**F**–**H**) The co-expression subnetworks of the top 10–20 hub genes of the medium purple 3, orange and dark magenta modules.

WGCNA can be used to explore the biological relevance between modules and target traits, and to identify core genes in the network. In order to investigate the molecular mechanisms underlying *M. glyptostroboides* seed aging, WGCNA was used to cluster and analyze gene expression data from 15 samples. After filtering out low-expressed genes, 10,882 genes were selected to determine the core genes that respond to seed aging. The aging response co-expression network was constructed by setting a threshold value of 15 and a merge cut height of 0.1851 in combination with the physiological indexes of different periods measured in the previous period. The network consisted of 15 modules: each branch represented a gene and each color represented a module. The smallest module contained 68 genes (royal blue), the largest module contained 6753 genes (turquoise), and 427 genes were classified as gray modules. The correlation analysis between modules and aging-related physiological indicators genes showed that the dark magenta module was highly correlated with GSH, orange with CAT, H_2_O_2_, and medium purple 3 with SOD, O_2_^·−^ (correlation > 0.8, *p* < 0.001) (Figure 10B). Therefore, GO functional enrichment analysis was performed on the above three core modules. The medium purple 3 module (3643 DEGs) was significantly enriched in the phosphorelay signal transduction system, peptide catabolic process, and programmed cell death (Figure 10C). The orange module (156 DEGs) was significantly enriched in the G-protein-coupled receptor signaling pathway, lipid catabolic process, and other processes (Figure 10D). The dark magenta module (938 DEGs) was significantly enriched in the regulation of protein modification process, amine metabolic process, ATP metabolic process, and other processes (Figure 10E).

Based on the eigengene connectivity (KME) values, the top 20 and 10 genes in the medium purple 3, orange and dark magenta modules were selected to construct co-expression subnetworks using Cytoscape_v.3.8.1 to identify potential candidates with significant contributions. The respiratory burst oxidase homologue (*Rboh*) gene, which encoded a protein involved in ROS production, had the highest KME value in the medium purple 3 module (Figure 10F). In addition, several genes related to plant hormone biosynthesis, signal transduction, and transcription factors, such as *GRF*, *AP2/ERF*, *PP2C*, *AAO3*, and *PIF3*, were also identified in the hub gene network. Gene *ACO*, which encoded an enzyme involved in ethylene biosynthesis, had the highest KME value in the orange module (Figure 10G), which also contained a *CAT* gene. Additionally, several other genes, such as *EEF2*, *EIF4A*, *GCAT*, *HSP70-1*, *metK*, *NDUFS1*, and *PGD*, were identified in this module. The heat shock transcription factor (*HSF*) gene had the highest KME value in the dark magenta module, which also contained other important genes such as *GLOS* and *CML* (Figure 10H). Six core genes were selected for qRT-PCR verification, and the results were consistent with the transcriptome data (Supplementary Figures S3 and S4), indicating that the transcriptome data accurately reflected transcript abundance in this study.

## 4. Discussion

### 4.1. Physiological Parameters of Seed Aging in M. glyptostroboides

Studies have found that seed vigor is a crucial factor in the difficult natural regeneration of *M. glyptostroboides* populations [34,35,36]. The study found that the germination percentage of fresh-dried *M. glyptostroboides* seeds was (58 ± 8.08)%, which increased to (70.67 ± 5.21)% after 2 days of aging. However, the germination percentage rapidly declined after 4 days of aging and died after 8 days of aging. Similar to the results of Liu et al., *M. glyptostroboides* seeds exhibited low vigor and a limited ability to sustain vigor [37]. Numerous studies have shown that, following the artificial aging treatment, there was a discernible downward trend in seed germination percentage, germination potential, germination index, and vigor index [38,39]. In this study, the germination percentage, germination potential, and vigor index of aged *M. glyptostroboides* seeds showed a significant decline, but there was no significant effect on seedling growth, which may suggest that seed germination limitations is not the main reason for the rare occurrence of undergrowth seedlings. It is noteworthy that after a short-term aging treatment (2 days), the mean germination percetage of *M. glyptostroboides* seeds were significantly higher than the control group (Figure 1A). Similar results were obtained for aged *Pyrus pyrifolia* seeds after a brief artificial aging treatment [40]. It may be that high-temperature and high-humidity conditions can promote the physiological post-ripening of *M. glyptostroboides* [41,42], and when the dry seeds absorb swelling under a high-humidity environment, a certain inducing effect is produced, causing an increase in antioxidant enzyme activity and thereby enhancing seed vigor and stress resistance [43], i.e., causing a seed priming effect. However, as the degree of aging further deepened, the seeds eventually lost all vigor and died [32]. 

Seed aging was a gradual and complex physiological and biochemical process involving numerous reactions [32]. The seed aging mechanism depended on temperature and humidity conditions, and it was believed that the seed aging mechanism was different at a low humidity and high temperature and high humidity. The cytoplasm enters a glassy state under low-humidity conditions, in which other processes lead to decreased vigor, and the biochemical characteristics were similar to seeds stored in a seed bank [9,10]. Under high-temperature and high-humidity conditions, the aging of forest plant seeds in the natural state can be simulated to a certain extent [44]. In the study of aging mechanism, most of the high-temperature and high-humidity conditions were used to accelerate the aging of seeds, and it is widely believed that the accumulation of ROS in aging seeds led to damage to phospholipids, lipid peroxidation, decreased antioxidant enzyme activity, inhibited RNA and protein synthesis, DNA degradation, and ultimately complete loss of seed vigor [45]. ROSs mainly include O_2_^·−^, H_2_O_2_, and ·OH, with O_2_^·−^ being generated in the process of electron leakage in the transfer chain and then being dismutated by SOD to generate H_2_O_2_ [32]. The imbalance of ROS content could have toxic effects on plants, while the antioxidant system of seeds could effectively remove excess ROS and harmful substances such as MDA, protecting cells from oxidative damage [32]. SOD is generally recognized as an important part of the first line of defense system against reactive oxygen species (ROS) [46,47,48]. In this study, SOD activity significantly decreased after aging treatment and remained at a low level, and the O_2_^·−^ content and production rate decreased significantly in the early stages of aging. This indicated that the first line of defense (SOD) of *M. glyptostroboides* seeds’ antioxidant system has strong antioxidant defense capabilities, dismutating O_2_^·−^ to generate H_2_O_2_ and gradually increasing the H_2_O_2_ and MDA content. There are three pathways for H_2_O_2_-centered ROS clearance, with CAT directly catalyzing the production of H_2_O and O_2_, APX clearing H_2_O_2_ through the AsA-GSH cycle, and GPX completely clearing it through the cycle [49]. CAT is a key enzyme in the antioxidant system. The main function of CAT is to remove ROS from plants. It is responsible for clearing H_2_O_2_ and regulating related signal pathways and mainly occurs in peroxisomes and glyoximes [50]. Liu et al. found that CAT played a protective role in the early stages of aging [25]. In this experiment, CAT and APX activity increased after 8 days of aging, which may have complementary or interactive effects on reducing oxidative damage [51]. However, APX had a higher affinity for H_2_O_2_ and was mainly responsible for fine-tuning its regulation [52]. MDA was a product of lipid peroxidation, while GPX was the main enzyme used to repair lipid peroxidation damage. Both can serve as important biological markers for oxidative stress and are used to measure the degree of membrane damage under stress [53,54,55]. The results showed that GPX activity gradually increased with the prolongation of seed aging time, similar to the trend of MDA content, indicating that severe damage is caused to lipids during seed aging. The AsA-GSH cycle is crucial for maintaining membrane protein structure stability during oxidative stress [56]. The GSH and AsA content of *M. glyptostroboides* seeds increased after 4 days of aging and then slowly decreased. At the same time, the MDA and H_2_O_2_ content gradually increased. Therefore, it was speculated that the failure of the AsA-GSH cycle system was a key factor in the process from seed aging to death.

### 4.2. Full-Length Sequences Identified by SMRT Sequencing in M. glyptostroboides Provided Resources for Studies of the Aging Stress Response

In the past decade, next-generation sequencing (NGS) technology has been widely used for gene discovery and research [57], and RNA-seq has become an important tool for evaluating the entire RNA expression pattern [58]. However, the sequencing fragment length of NGS is usually between 50 and 300 bp, which produces hundreds of thousands of transcripts for large genome species such as *M. glyptostroboides* that lack a reference genome [59], greatly limiting the physiological and genetic mechanism studies of this species. Single-molecule real-time sequencing technology is a third-generation sequencing technology used to obtain longer transcripts. Full-length sequencing could identify the complete structure of single transcripts, especially lncRNA, which uniquely reveals the complexity of the transcriptome and effectively compensates for the shortcomings of second-generation sequencing technology [60]. In this study, a total of 32.18 Gb of raw data were obtained using SMRT and, after cluster analysis, FLNC sequence correction and redundant sequence removal, we obtained 42,189 unigenes with an average length of 2814 bp. This is far superior to previous transcriptome studies of loquat that only used second-generation sequencing technology. Our research results were similar to those of studies in *Fragaria vesca* [61], *Medicago sativa* [62], and *Rhododendron lapponicum* [63], which showed that SMRT sequencing was an effective way to obtain reliable full-length transcriptome sequence information in plants. Although *M. glyptostroboides* was not a model plant, a functional annotation of the 42,189 unigenes was obtained in this study based on existing databases of reference plants. A total of 40,443 genes were annotated in 5 databases, accounting for 95.86% of the annotated genes, and 21,397 genes were simultaneously annotated in all 5 databases, accounting for 50.71% of the total. The annotation of the NR database revealed a close kinship between *M. glyptostroboides* and North American spruce. In the analysis of gene structure, the predicted CDS results showed that the longer the transcript sequence, the fewer the copies in the cell, which was consistent with previous results in other species analyzed by full-length transcriptome [63,64]. In this study, we used third-generation sequencing technology to conduct full-length transcriptome sequencing analysis of *M. glyptostroboides*, which enriched the transcriptome database of this species and provided data support for research on its growth and development mechanisms, metabolic regulation, key functional gene screening, and genetic diversity analysis.

### 4.3. DEGs in Response to Aging Stress

Aging is a complex network regulation process, and the physiological and biochemical changes in the early stages of seed aging involve the expression regulation of thousands of genes [65]. However, with futher aging, gene expression regulation network interactions significantly decrease. In this study, it was found that as the seed aging progressed, the number of genes with FPKM ≤ 0.3 gradually increased, which was similar to the results of most transcriptome and proteomic studies on seed aging [66,67,68]. When plants were subjected to stress, a large number of genes were upregulated or downregulated in response to internal physiological changes [69,70]. In this study, the identified DEGs gradually decreased, and the significant DEGs in S4 vs. S6 were much less than those in S4 vs. S2, indicating that the period between S4 and S6 is a critical period for seed resistance to aging. In addition, all DEGs were divided into 10 clusters through K-mer analysis, and these gene clusters participated in multiple functions with temporal and spatial expression specificity.

Mitochondria are important organelles that produce adenosine triphosphate (ATP) during cellular respiration; they are also the main sites of ROS production during seed aging [71]. Studies have found that seed aging affects the respiratory pattern of mitochondria, exhibiting a complex and variable respiratory pattern, including COX, alternative oxidase (AOX), and uncoupling protein (UCP) pathways [45]. The release of cytochrome c may be a major reason for the inhibition of the mitochondrial electron transport chain, further increasing ROS accumulation, and causing changes in cellular components that damage cells and accelerate seed aging [72]. In this study, it was found that cytochrome c oxidase, as a marker of mitochondrial respiratory enzymes, was gradually upregulated during aging, indicating that the inhibition of electron transport and respiration occurs due to the release of cytochrome c, resulting in the accumulation of ROS. AsA and GSH play important roles in regulating the mitochondrial redox state. AsA is involved in regulating gene expression, enzyme activity, and cell signaling in redox regulation [32]. Changes in mitochondrial AsA synthesis may regulate communication between the plastid and mitochondria [73]. A lack of GSH in the mitochondria causes mitochondrial damage, changes the synthesis of thiol proteins, and alters mitochondrial redox regulation [74]. GSH is mainly synthesized in the cytoplasm and plastid; the level of mitochondrial GSH depends on the transport of GSH into the mitochondria [75]. The level of mitochondrial GSH is highly dependent on the activity of glutathione reductase (GR), which reduces GSH to its oxidized form, glutathione disulfide (GSSG) [76]. In the antioxidant system of this study, the expression of *CAT* and *POD* genes is very active, while the trend of *AsA* and *GSH* levels and the expression of *GR*, *APX*, and *MDHR* genes are similar, with all gradually decreasing, further confirming the failure of the AsA-GSH cycle and ultimately leading to the accumulation and overflow of ROS. The accumulation of ROS induces membrane lipid peroxidation, which, in turn, reduces the integrity of the cell membrane [77]. Phospholipase D (*PLD*) is a key enzyme that catalyzes the hydrolysis of membrane phospholipids, and lipoxygenase (*LOX*) further catalyzes the generation of ROS and oxygen free radicals from the degradation of membrane phospholipids by *PLD*, ultimately leading to the destruction of the membrane phospholipid bilayer structure [78]. In this study, most *PLD* genes were upregulated in the late aging period after aging treatment, while most of the *LOX* genes were only significantly expressed in the control group. It is probable that most genes are expressed in the early stages of aging. The ROS content did not increase significantly, but the upregulation of *PLD* gene expression led to membrane lipid degradation and damage. In addition, the physiological indicators showed that the content of MDA continuously increased with age, indicating that lipid peroxidation caused oxidative damage.

It has been demonstrated that seed-aging-induced oxidative stress led to the accumulation of protein misfolding and organelle dysfunction, ultimately resulting in programmed cell death [8]. Chen et al. found that the artificial aging of pea seeds can induce ERs, as evidenced by the upregulation of the ERs’ marker protein BiP2 [79]. In this study, we observed a significant upregulation of *BiP* expression in *M. glyptostroboides* seeds after two days of aging, indicating the occurrence of ERs during the aging process. The proper formation of disulfide bonds was crucial to protein maturation and stability [80]. In eukaryotic cells, the ER oxidoreductase family, including PDI and ER protein 72 (ERp72), catalyzed protein oxidation and folding, with PDI acting as an enzymatically active molecular chaperone [81]. Protein folding and refolding on the ER were energy-intensive processes, and the misfolding of proteins consumed ATP, which may stimulate mitochondrial oxidative phosphorylation. We detected the upregulation of four differentially expressed PDIs and ATPases during seed aging, which increased ATP and ROS production. Accumulation of unfolded proteins in the ER can promote the release of Ca^2+^ into the cytoplasm and increase ROS production in the mitochondria [82]. Interference with mitochondrial respiration significantly reduced ROS accumulation induced by unfolded proteins [83]. Mitochondrial ROS production may, in turn, enhance the ERs response, leading to the further accumulation of mitochondrial ROS. This represented a potential signaling mechanism for the interplay between ERs-induced ROS and mitochondrial dysfunction [84]. We speculated that ROS production and accumulation during seed aging alter the oxidoreductive environment of the ER, leading to ERs. During the process of promoting proper disulfide bond formation in unfolded and misfolded proteins, the ER may also generate large amounts of ROS. ERs signaling affects mitochondria, further exacerbating mitochondrial ROS production and leading to further increases in ROS levels in the cell.

It has been reported that Arabidopsis alleviated ERs by upregulating potential yeast mammalian homologs of ERAD components [85]. Liu et al. demonstrated that, under salt stress, unfolded proteins rapidly accumulate in the ER and induce the UPR. Defects in HRD3A, a component of the HRD3/HRD1 complex in the ERAD pathway, led to changes in the UPR and retention of ERAD substrates in plant cells, increasing plant sensitivity to salt stress [86]. In this study, significant expression changes were observed in UbcH5, E3 ubiquitin ligase Hrd1, and Doa10, indicating that the ERAD pathway can respond to artificial aging treatment in *M. glyptostroboides* seeds. The ERAD pathway required the participation of numerous proteins with different functions, and ER mannosidase I (ERManI) and ER degradation-enhancing α-mannosidase-like protein (EDEM) played important roles in substrate recognition [84]. EDEM was a transmembrane protein localized in the ER and served as a protein receptor and oligosaccharide-binding site, releasing misfolded proteins from the Calnexin/Calreticulin cycle and promoting their degradation [87]. Decreased levels of EDEM in cells led to the accumulation of misfolded proteins in the ER, affecting the efficiency of normal protein folding and assembly [88]. In this study, the downregulation of EDEM expression was detected during seed aging, which may delay the release of misfolded proteins from the Calnexin/Calreticulin cycle, inhibit the ERAD pathway, and cause the accumulation of misfolded proteins in the ER, leading to cell damage and decreased seed vigor. *UbcH5* was found to be significantly upregulated in this study, but further research was needed to determine its role as a major component of the ERAD complex. *HRD1*, a ubiquitin ligase, was an important component of the ERAD pathway in plants and was responsible for the degradation of bri1-5 and bri1-9 proteins [89]. This study showed that 8 *Hrd1* and *Doa10* genes were differentially expressed, with the highest expression observed on day 2 of aging, indicating that the ERAD complex actively responded to protein ubiquitination in the early stages of seed aging. The *ATPase P97*, together with auxiliary factors such as *Ufd1* and *Npl4*, promoted the ubiquitination and dissociation of substrate proteins from the ER membrane structure. Ubiquitinated proteins were then transported to the proteasome with the help of radiation-sensitive protein RAD23 and Dsk2 protein [90]. The upregulation of *P97* was detected in this study, indicating the accumulation of misfolded proteins during the transport and release process, while the downregulation of *UBX* and *DUB* affected the process of substrate proteins entering the 26S proteasome.

In summary, a large number of DEGs participated in the recognition, transport, and degradation of misfolded proteins during seed aging. Although the ERAD pathway responded actively to ER stress, the downregulation of the key gene *EDEM* may reduce the degradation rate of misfolded proteins, leading to their accumulation and damage to cells. The repair of misfolded proteins required the oxidative phosphorylation pathway to provide a large amount of energy, which further increased ROS production. The failure of the AsA-GSH cycle exacerbated ROS leakage, leading to the further accumulation of misfolded proteins and ultimately resulting in programmed cell death through nucleic acid degradation. Therefore, it is suggested that the obstruction of the ERAD pathway, the release of COX enzymes, and the failure of the AsA-GSH cycle may be key factors leading to the loss of seed vigor, which requires further experimental validation.

### 4.4. Identification of Hub-Genes Associated with Scavenging ROS in Seeds

WGCNA was a powerful tool widely used in physiological mechanism research [91,92]. Genes that were highly connected within a module were considered to be central genes, which were believed to constitute the backbone of the network and play a crucial role in specific physiological processes [93]. To establish the association between genes and traits and further analyze the regulatory mechanism, WGCNA combined DEGs and physiological indicators of seed aging, identifying 15 modules, among which dark magenta, medium purple 3, and orange modules were considered key modules. In the medium purple 3 module, the expression of repiratory burst oxidase homologue (*Rboh*) was highly correlated with the content and production rate of SOD and O_2_^·−^. *Rboh* catalyzed the production of ROS by O_2_^·−^ and NADPH [94]. Studies have shown that *Rbohs* plays an important role in plant biotic and abiotic stress, mainly by producing ROS to induce host defense genes and phosphorylate protein kinases, activate transcription factors, and activate ion transport systems [94]. ROS plays a dual role in plant stress response, acting as a toxic substance causing oxidative damage and as a signaling molecule mediating numerous biological processes [95]. In this module, the expression pattern of *Rbohs* gradually decreased with the aging process. The genes in this module were significantly enriched in GO functions such as the phosphorelay signal transduction system, peptide catabolic process, and programed cell death, indicating that the phosphorelay signal transduction systems that are dependent on *Rbohs*, such as *AP2/ERF, PP2C*, and *PIF3*, were inhibited. *Rbohs* were identified as hub genes related to the content and production rate of SOD and O_2_^·−^, further demonstrating the reliability of the hub-genes screened by WGCNA in this study.

During seed aging, changes in endogenous hormones such as a decrease in GA3 and IAA levels and an increase in ABA levels accompanied the process, leading to hormonal imbalances, an important factor affecting seed aging and deterioration [96]. Among them, an increase in ethylene release played an important role in reducing the degree of seed aging [96]. As a key enzyme in the ethylene biosynthesis pathway, 1-aminocyclopropane-1-carboxylic acid (ACC) was widely used as a representative of ethylene, and almost all plant tissues can easily convert this into ethylene [97]. In addition, studies have shown that an increase in ethylene expression often increases CAT activity [98]. In this study, the orange module was highly correlated with CAT activity, and ACO was identified as the hub gene in this module, with CAT being identified as one of the top 10 hub genes. This suggests that ethylene may play a significant role in seed aging by regulating antioxidant enzyme activity.

HSPs act as molecular chaperones and play an important role in protein stability by participating in peptide folding to protect against oxidative damage [99]. Overexpression of small HSPs (*sHSPs*) in plants has been shown to enhance stress resistance [100]. For example, overexpression of the *NnHSP17.5* gene in lotus in *Arabidopsis thaliana* enhanced seed germination and seedling heat tolerance, indicating that HSPs may be involved in oxidative-stress-related respondes during germination [101]. Heat shock transcription factor (*Hsf*) is a transcription factor that recognizes heat shock elements in the upstream promoter regions of *Hsp* genes and transcribes them following activation by heat stress [102]. The overexpression of *Hsf* in *Arabidopsis* seeds increases the expression of *HSPs* and enhances anti-aging ability [103]. Therefore, *Hsf* participates in the stress response by regulating the expression of heat shock proteins. In addition, the calcium–calcium-binding protein (Ca^2+^-CaM) pathway plays a critical role in signal perception, transduction, transcriptional regulation, and functional protein expression. Aging stimuli caused an increase in intracellular Ca^2+^ concentration through an unknown receptor, which activates Ca^2+^-CaM or promotes the expression of *CaM* genes to activate *HSF*, which, in turn, activates the expression of heat shock genes [104]. The accumulation and expression of *HSP* structures protects against damage caused by stress, thereby reducing the damage to plant seeds caused by aging stress. In this study, we found that the genes in the dark magenta module were significantly enriched in the “regulation of protein modification process” GO term, and *HSF* was identified as the hub gene of this module. We also found three CaM-like (CML) genes as hub genes, which showed consistent expression trends with *HSF*. The significant enrichment of the “regulation of protein modification process” GO term in the dark magenta module indicates that *CML* and *HSF* play an important role in repairing protein oxidative damage caused by seed aging by regulating the accumulation of *HSPs*.

## Figures and Tables

**Table 1:** Changes of germination potential, germination index, vigor index, root length, seedling height and seedling dry/fresh weight with artificial aging treatment time.

Aging Time (d)	Germination Percentage (%)	Germination Potential (%)	Germination Index (%)	Vigor Index (%)	Root Length (mm)	Seedling Height (mm)	Fresh Weight (mg)	Dry Weight (mg)
0	58.00 ± 5.71 ^b^	54 ± 5.29 ^a^	12 ± 1.32 ^a^	59.54 ± 7.40 ^a^	12.79 ± 1.55 ^a^	36.63 ± 1.08 ^a^	18.55 ± 0.50 ^a^	2.01 ± 0.02 ^a^
2	70.67 ± 3.68 ^a^	48 ± 3.46 ^a^	11.46 ± 0.53 ^a^	63.16 ± 4.63 ^a^	17.90 ± 1.15 ^a^	37.09 ± 0.58 ^a^	20.13 ± 0.71 ^a^	2.04 ± 0.06 ^a^
4	33.33 ± 4.11 ^c^	10 ± 2.31 ^b^	4.64 ± 0.84 ^b^	22.86 ± 4.63 ^b^	12.59 ± 1.13 ^a^	36.39 ± 0.52 ^a^	20.59 ± 0.54 ^a^	1.94 ± 0.02 ^ab^
6	9.33 ± 1.88 ^d^	0 ^c^	1.23 ± 0.33 ^c^	5.55 ± 1.41 ^c^	12.03 ± 1.31 ^a^	33.84 ± 1.12 ^a^	21.51 ± 1.22 ^a^	1.82 ± 0.04 ^b^
8	0 ^e^	—	—	—	—	—	—	—

Note: “—” means no normal seedling. There are no significant differences between data marked with the same lowercase letters in the same column (*p* > 0.05).

## Data Availability

Not applicable.

## Supplementary Materials

The following supporting information can be downloaded at: https://www.mdpi.com/article/10.3390/antiox12071353/s1, Table S1: Primers used for quantitative real-time RT-PCR analysis. Table S2: Summary of reads from third-generation, long-read sequencing. Table S3: all unigene functional annotation. Table S4: GO and KEGG functional annotation of all genes. Table S5: RNA-seq mapping full-transcription information. Table S6: Go functional annotation of DEGs in S2 vs. S0. Table S7: Go functional annotation of DEGs in S4 vs. S2. Table S8: Go functional annotation of DEGs in S6 vs. S4. Table S9: Go functional annotation of DEGs in S8 vs. S6. Table S10: Differentially expressed genes related to protein processing in endoplasmic reticulum pathways in the five aging stages of *M. glyptostroboides* seeds. Table S11: Differentially expressed genes related to the oxidative phosphorylation pathways in the five aging stages of *M. glyptostroboides* seeds. Table S12: Differentially expressed genes related to the ROS scavenging system in the five aging stages of *M. glyptostroboides* seeds. Figure S1: RNA integrity detection. Figure S2: FPKM expression of samples in different periods. Figure S3: qRT-PCR verification of the expression pattern according to the RNA-seq data. Figure S4: qPCR fusing curve.
