# Supplementary material for: Full-Length Transcriptome Sequencing Reveals the Molecular Mechanism of Metasequoia glyptostroboides Seed Responding to Aging
Source: Antioxidants (Basel). 2023 Jun 27;12(7):1353. doi: 10.3390/antiox12071353 (PMC10376015; doi:10.3390/antiox12071353)
Supplement: Supplementary file 1 [file antioxidants-12-01353-s001.zip › Supplementary Figure.pdf]

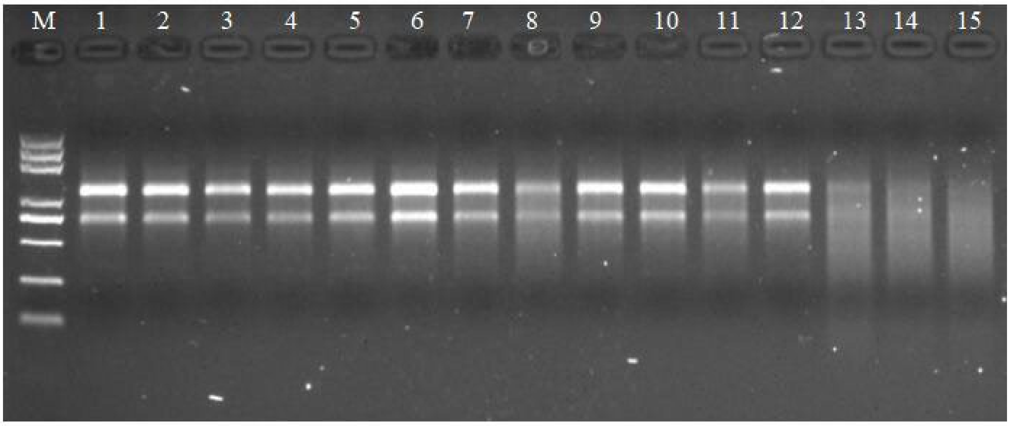

Figure S1. RNA agarose gel electrophoresis pattern,1-15 Wells are S0-1, S0-2, S0-3, S2-1, S2-2, S2-3, S4-1, S4-2, S4-3, S6-1, S6-2, S6-3, S8-1, S8-2, S8-3.

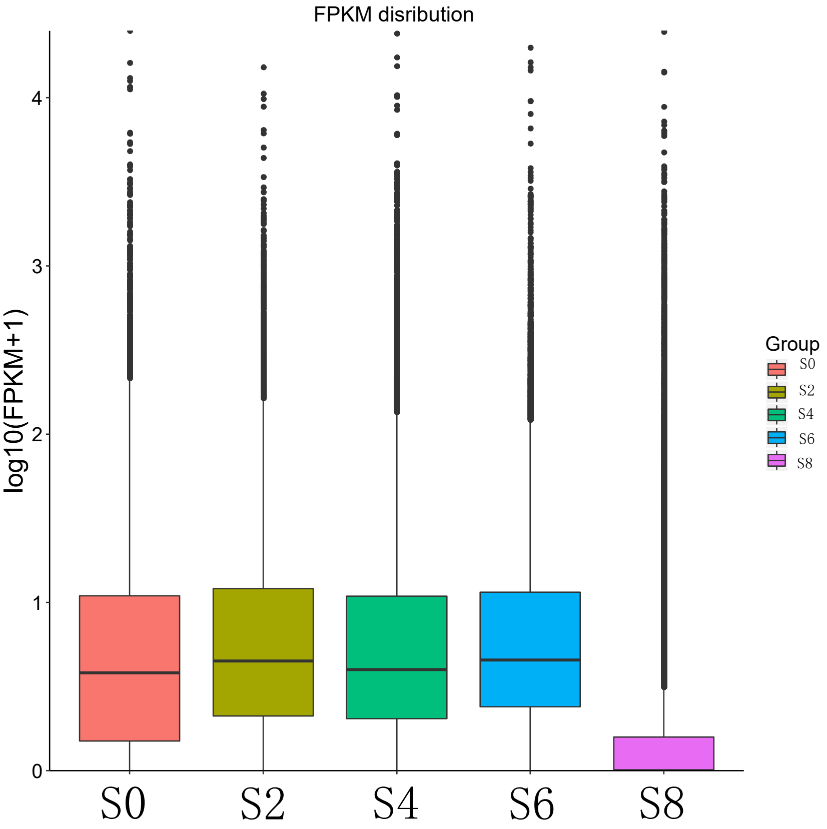

Figure S2. FPKM expression of samples in different periods

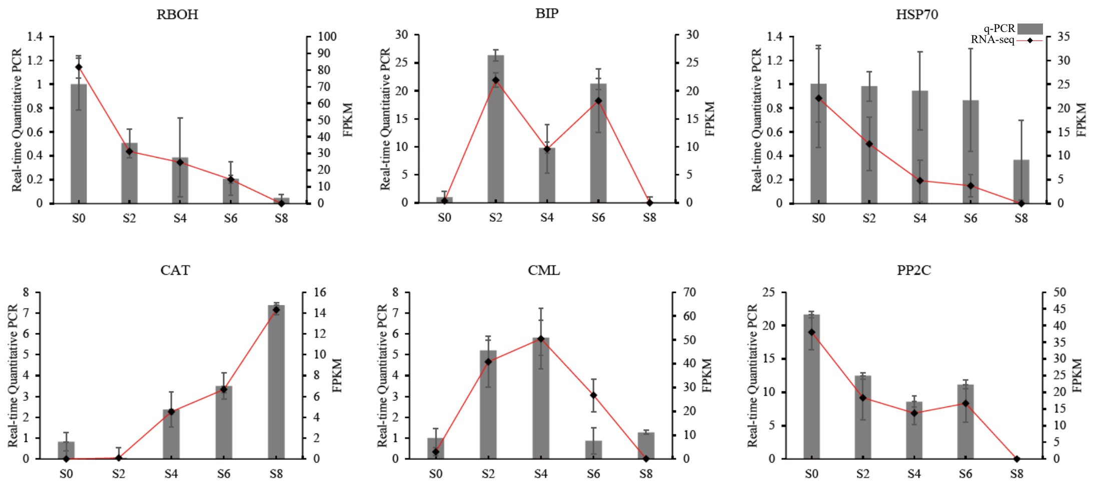

Figure S3. qRT-PCR verification of the expression pattern according to the RNA-seq data.

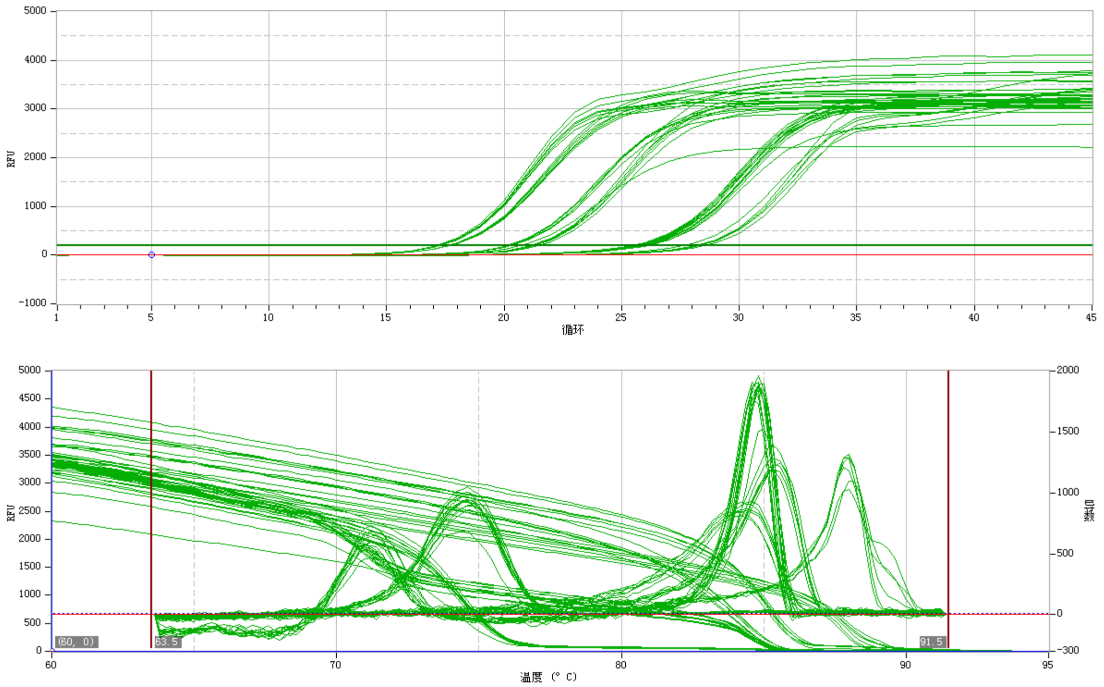

Figure S4 qRT-PCR fusing curve
